# Supplementary material for: Causal Relationship between Aging and Anorexia Nervosa: A White-Matter-Microstructure-Mediated Mendelian Randomization Analysis
Source: Biomedicines. 2024 Aug 16;12(8):1874. doi: 10.3390/biomedicines12081874 (PMC11351342; doi:10.3390/biomedicines12081874)
Supplement: Supplementary file 1 [file biomedicines-12-01874-s001.zip › Supplementary Figure/Figure S4/Singe SNP plot of MR analysis for TL to ubm-b-1477.pdf]

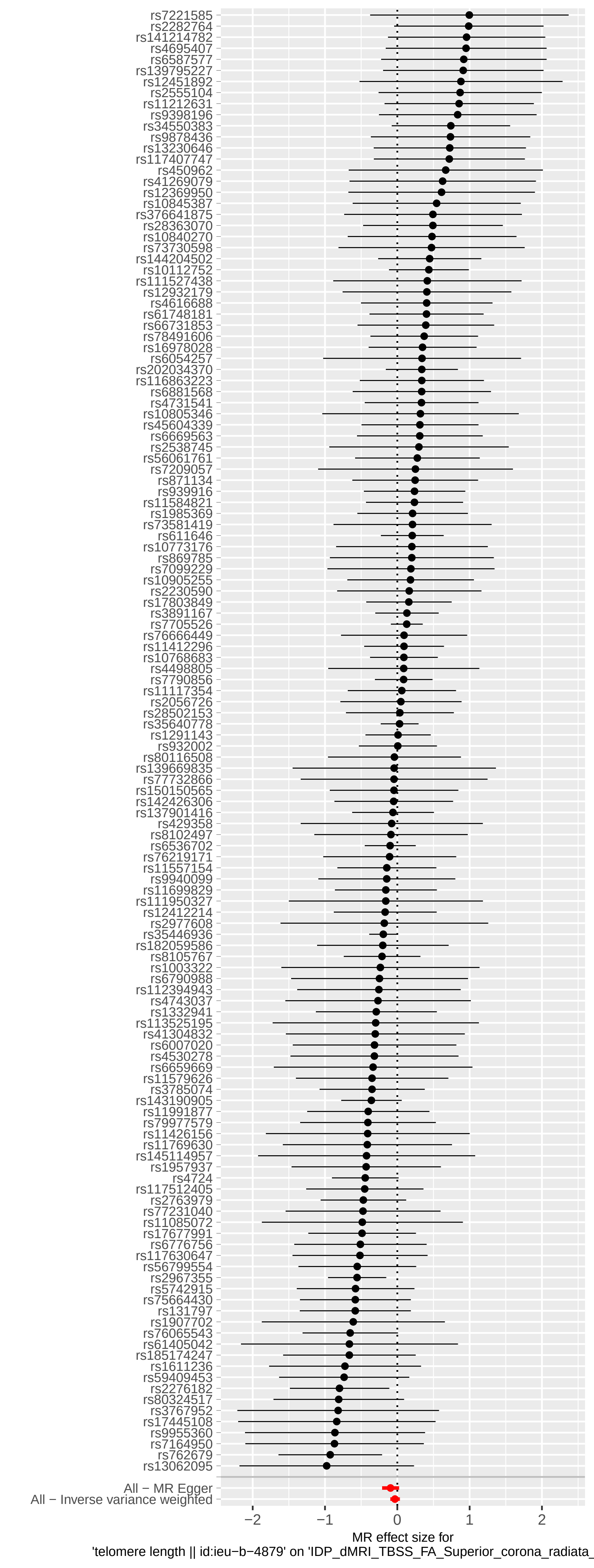
rs7221585
rs2282764
rs141214782
rs4695407
rs6587577
rs139795227
rs12451892
rs2555104
rs11212631
rs9398196
rs34550383
rs9878436
rs13230646
rs117407747
rs450962
rs41269079
rs12369950
rs10845387
rs376641875
rs28363070
rs10840270
rs73730598
rs144204502
rs10112752
rs111527438
rs12932179
rs4616688
rs61748181
rs66731853
rs78491606
rs16978028
rs6054257
rs202034370
rs116863223
rs6881568
rs4731541
rs10805346
rs45604339
rs6669563
rs2538745
rs56061761
rs7209057
rs871134
rs939916
rs11584821
rs1985369
rs73581419
rs611646
rs10773176
rs869785
rs7099229
rs10905255
rs2230590
rs17803849
rs3891167
rs7705526
rs76666449
rs11412296
rs10768683
rs4498805
rs7790856
rs11117354
rs2056726
rs28502153
rs35640778
rs1291143
rs932002
rs80116508
rs139669835
rs77732866
rs150150565
rs142426306
rs137901416
rs429358
rs8102497
rs6536702
rs76219171
rs11557154
rs9940099
rs11699829
rs111950327
rs12412214
rs2977608
rs35446936
rs182059586
rs8105767
rs1003322
rs6790988
rs112394943
rs4743037
rs1332941
rs113525195
rs41304832
rs6007020
rs4530278
rs6659669
rs11579626
rs3785074
rs143190905
rs11991877
rs79977579
rs11426156
rs11769630
rs145114957
rs1957937
rs4724
rs117512405
rs2763979
rs77231040
rs11085072
rs17677991
rs6776756
rs117630647
rs56799554
rs2967355
rs5742915
rs75664430
rs131797
rs1907702
rs76065543
rs61405042
rs185174247
rs1611236
rs59409453
rs2276182
rs80324517
rs3767952
rs17445108
rs9955360
rs7164950
rs762679
rs13062095
All − MR Egger
All − Inverse variance weighted
−2
−1
0
1
2
MR effect size for
'telomere length || id:ieu−b−4879' on 'IDP_dMRI_TBSS_FA_Superior_corona_radiata_
